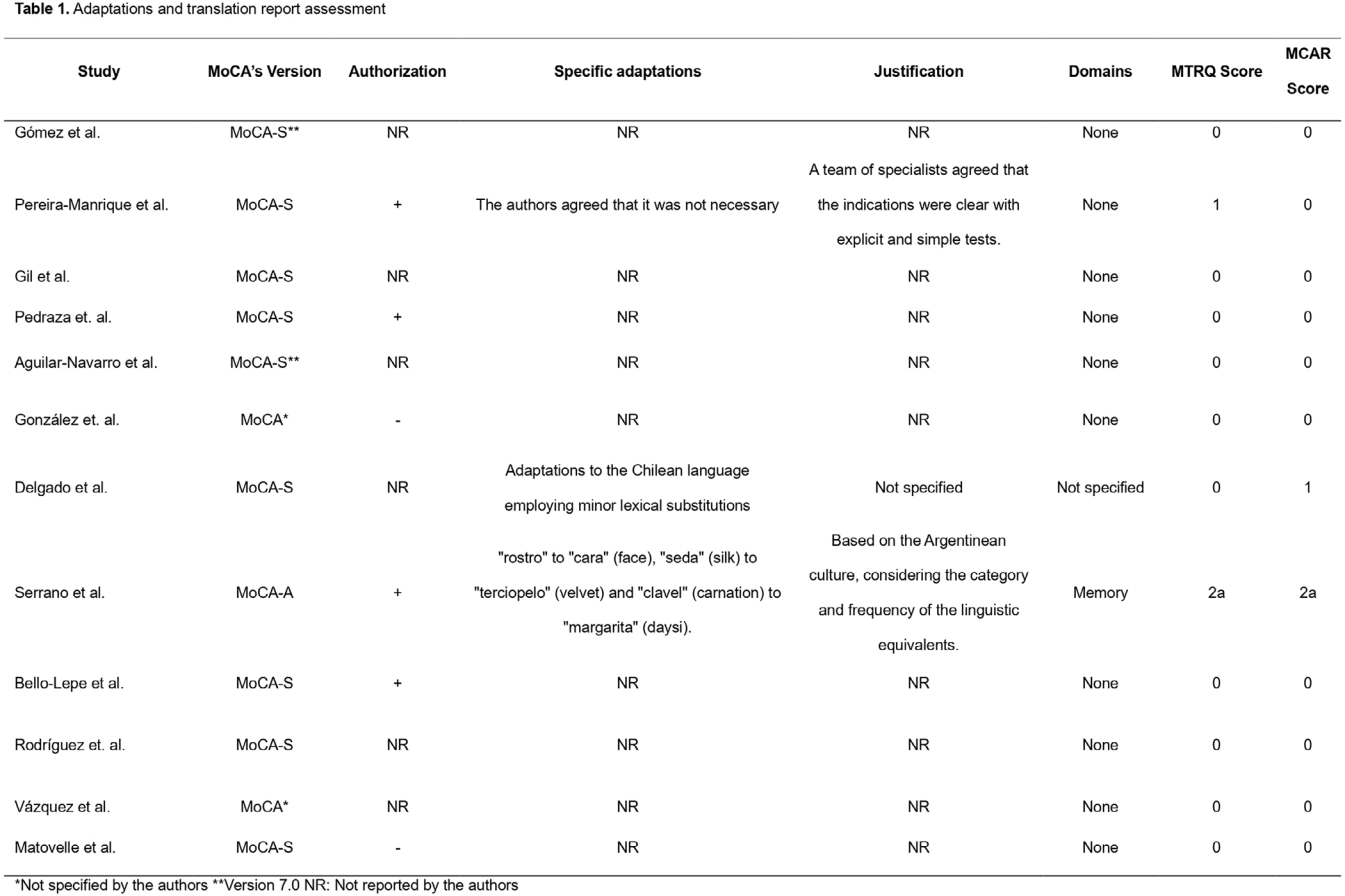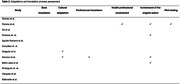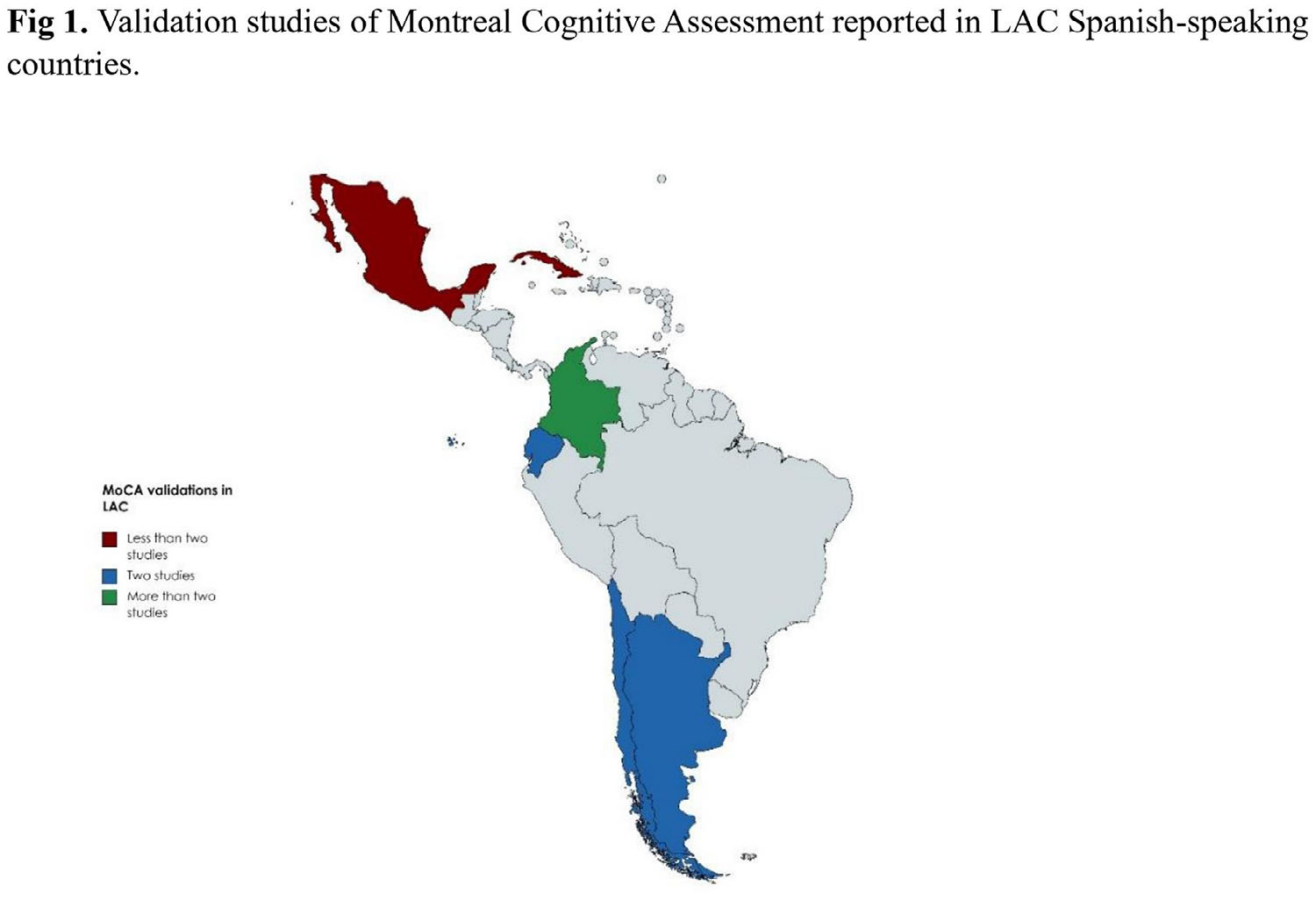# Montreal Cognitive Assessment in Spanish‐speaking countries in Latin America and the Caribbean: A Systematic Review

**DOI:** 10.1002/alz.091660

**Published:** 2025-01-09

**Authors:** Diego Bustamante‐Paytan, Angie Diaz‐Mejia, Gianinna L. Saenz‐Orihuela, Enrique Moncada‐Mapelli, Leslie Salazar, Rosa Montesinos, Adrian Noriega de la Colina, Nilton Custodio

**Affiliations:** ^1^ Cognitive Impairment Diagnosis and Dementia Prevention Unit, Peruvian Institute of Neurosciences, Lima, Lima Peru; ^2^ Universidad de San Martin de Porres, Lima, Lima Peru; ^3^ Universidad Ricardo Palma, Lima, Lima Peru; ^4^ Universidad Científica del Sur, Lima, Lima Peru; ^5^ Department of Neurology and Neurosurgery, McGill University, Montreal, QC Canada; ^6^ The Montreal Neurological Institute, McGill University, Montreal, QC Canada

## Abstract

**Background:**

The prevalence of dementia in Low and Middle‐Income Countries (LMICs), particularly in Latin America and the Caribbean (LAC), is projected to triple by 2050. The Montreal Cognitive Assessment (MoCA) is widely used for cognitive evaluation, but its uniform application in LAC is questionable, especially due to cultural and linguistic diversity of the Spanish‐speaking LAC countries.

**Method:**

A systematic literature search was conducted across seven databases, supplemented by a comprehensive review using Google Scholar to identify relevant grey literature. Studies validating or culturally adapting the MoCA, conducted in Spanish‐speaking institutions in the LAC region, with available psychometric measures and comparisons to gold‐standard diagnostic criteria, were considered for inclusion. Fourteen studies were identified that validated the MoCA in LAC. Twelve studies meeting inclusion criteria were analyzed using the Manchester Cultural Adaptation Questionnaire and Manchester Translation Reporting Questionnaire. The adaptation process and psychometric reports were thoroughly assessed.

**Result:**

Among all reviewed articles, 12 detailed MoCA validation across LAC populations (Fig. 1). Only two reported significant adaptations to the original version, with one study providing a detailed cultural rationale (Table 1). The adaptation process assessment revealed limited reporting on translation steps (Table 2). Concerning the psychometric report, internal consistency, criterion validity, and construct validity were the most frequently addressed aspects across all studies. In all studies where low‐educated populations were evaluated, educational level was reported to bias the results. We identified a lack of standardized criteria for definition of illiteracy across the studies.

**Conclusion:**

This systematic review identified limited reporting on translation steps emphasizes the need for standardized processes in future adaptations. We recommend cultural awareness during MoCA administration in the region. Future studies must address appropriate cut‐off scores based on educational level adjustments in LAC countries.